# Industrial Melanism in the Peppered Moth Is Not Associated with Genetic Variation in Canonical Melanisation Gene Candidates

**DOI:** 10.1371/journal.pone.0010889

**Published:** 2010-05-28

**Authors:** Arjen E. van't Hof, Ilik J. Saccheri

**Affiliations:** School of Biological Sciences, University of Liverpool, Liverpool, United Kingdom; American Museum of Natural History, United States of America

## Abstract

Industrial melanism in the peppered moth (*Biston betularia*) is an iconic case study of ecological genetics but the molecular identity of the gene determining the difference between the typical and melanic (*carbonaria*) morphs is entirely unknown. We applied the candidate gene approach to look for associations between genetic polymorphisms within sixteen *a priori* melanisation gene candidates and the *carbonaria* morph. The genes were isolated and sequence characterised in *B. betularia* using degenerate PCR and from whole-transcriptome sequence. The list of candidates contains all the genes previously implicated in melanisation pattern differences in other insects, including *aaNAT*, *DOPA-decarboxylase*, *ebony*, *tan*, *tyrosine hydroxylase*, *yellow* and *yellow2* (*yellow-fa*). Co-segregation of candidate gene alleles and *carbonaria* morph was tested in 73 offspring of a *carbonaria* male-typical female backcross. Surprisingly, none of the sixteen candidate genes was in close linkage with the locus controlling the *carbonaria*-typical polymorphism. Our study demonstrates that the ‘*carbonaria* gene’ is not a structural variant of a canonical melanisation pathway gene, neither is it a *cis*-regulatory element of these enzyme-coding genes. The implication is either that we have failed to characterize an unknown enzyme-coding gene in the melanisation pathway, or more likely, that the ‘*carbonaria* gene’ is a higher level *trans*-acting factor which regulates the spatial expression of one or more of the melanisation candidates in this study to alter the pattern of melanin production.

## Introduction

Industrial melanism in the peppered moth *Biston betularia* remains the textbook example of a rapid evolutionary response to a dramatically altered environment. Essentially unknown before the first official recording from 1848 Manchester (England), by the turn of the 19^th^ century the black (*carbonaria*) morph had largely if not entirely replaced the light-speckled (typical) form in the most polluted parts of UK [1]. Following 1960s legislation to control smoke pollution, reverse selection has reduced *carbonaria* to a rarity [2]. Yet, despite its celebrity status within evolutionary biology, the molecular genetic and developmental control of the *carbonaria*-typical polymorphism is unknown. All that is known on this topic is that the trait is controlled by a dominant allele at a single Mendelian locus [3].

One approach to identifying genetic switches is to look for associations between the trait of interest and molecular genetic variation within known genes in the biochemical pathway. This candidate gene approach has been applied successfully to discover the genetic basis of melanism in mammals and other vertebrates, implicating several different mutations within the melanocortin-1-receptor gene (*Mc1r*) [4], [5]. In arthropods, the process of melanisation is variously used for purposes of crypsis, sexual signalling [6], and thermoregulation [7] but also plays a major role in immune response [8], wound healing and cuticular hardening [9]. Melanisation as a visual signal in adults is mainly a genetically determined developmental trait, whereas melanisation as defence is an induced (phenotypic) response.

**Figure 1:**
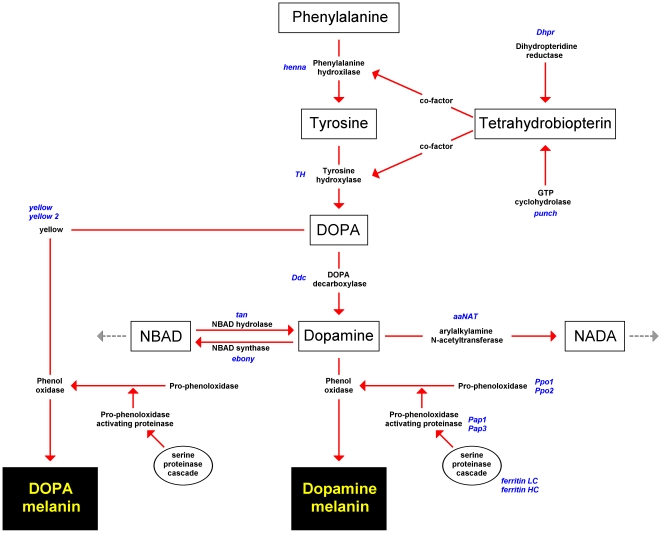
Insect melanisation biochemical pathway. The initial substrate, intermediate metabolites and two types of melanin are shown in rectangles. The enzymes that catalyze the different reactions are in black and the genes that code for these enzymes are italicized in blue. The genes in this figure correspond with the candidate genes that have been isolated and examined. The serine proteinase cascade is represented as a single cluster of events. A more comprehensive representation of this cascade is produced in Cerenius and Söderhäll [8]. The interrupted arrows pointing away from NBAD and NADA indicate that these are not the final metabolites. (Figure adapted from De Gregorio *et al.*, True, and Futahashi & Fujiwara [16], [17], [20]).

The biosynthesis of melanin as a pigment has been well characterized, predominantly in *Drosophila* [10], [11] and Lepidoptera [12], [13], [14], [15], [16], [17]. The universal mechanism which forms the core of the melanisation pathway involves a conserved pattern of substrate substitutions catalysed by a number of distinct genes. It begins with the two-step transformation of phenylalanine to tyrosine, then DOPA (Figure 1). These two transformations are mediated by phenylalanine hydroxylase (*PAH* or *henna*) and tyrosine hydroxylase (*TH or pale*) respectively. Tetrahydrobiopterin (BH_4_) is an essential cofactor (electron donor) for *henna* and *TH*, and is in turn dependent on guanosine triphosphate-cyclohydrolase I (*GTPCHI* or *punch*) and dihydropteridine reductase (*Dhpr*). DOPA is subsequently transformed into either DOPA-melanin or dopamine-melanin. The formation of DOPA-melanin is catalyzed by one or more members of the *yellow* gene family and by phenoloxidase (*PO*). Transition from DOPA to dopamine-melanin involves the formation of dopamine by DOPA decarboxilase (*Ddc*) and subsequently dopamine-melanin by *PO*. Dopamine is also utilized by NBAD synthase (*ebony or BAS*) and arylalkylamine N-acetyl transferase (*aaNAT*) to produce pigments other than melanin. In the swallowtail butterfly, *Papilio glaucus*, this alternative use of substrate lowers the dopamine titer to levels that prevent wing melanisation [18]. NBAD hydrolase (*tan*) catalyses the reaction in the opposite direction to *ebony*, increasing Dopamine concentration [19].

Immune-defence melanisation in arthropods is triggered by an intrusion of microbes, fungi, or other foreign objects, or by physical damage (wounds) [8], [20], [21]. Melanin encapsulates and immobilizes these harmful intrusions, or forms a barrier that replaces damaged cuticle, and at the same time, quinone intermediates that are highly toxic to pathogens are released as a by-product of the enzymatic processes involved [8]. The immune response proceeds through a series of biochemical transformations of proteolytic enzymes in a process named the serine proteinase cascade (reviewed in [8]). This cascade has branches with specific genes corresponding with the different types of intrusions and its activity can be locally reduced or blocked by serine proteinase inhibitors (serpins) [20], [22]. The cascade ultimately feeds into the core of the melanisation pathway (Figure 1) by transforming pro-phenol oxidase (*Ppo*) to *PO* by pro-phenoloxidase activating proteinases (*Pap* or *Ppae*) [23], [24], [25]. This complexity of gene interactions makes it difficult to explore all representatives of the serine proteinase cascade as melanisation candidate genes. Moreover, it is often not possible to identify orthologs of genes in different species by means of protein sequence comparison alone, because serine proteinases and serpins belong to very large gene families in which highly similar members (i.e. paralogs) often have completely unrelated functions [26], [27], [28], [29]. Ferritin, which plays a role in the serine proteinase cascade in *Manduca sexta* [30], is an exception because it is coded by two distinct subunits that are not easily confused with paralogs.

The premise of the candidate gene approach as a means of identifying causal mechanisms, rather than downstream consequences, is that the functional polymorphism resides either within the candidate gene itself, or is closely linked to it (e.g. *cis*-regulatory element). The current study examines 16 genes as potential candidates controlling the *carbonaria*-typical polymorphism in *B. betularia*. The genes that are involved with pigment-related melanisation, i.e., those in the core of the pathway, are expected to be more relevant with regard to industrial melanism than those that are triggered by an immune response. A number of these pigment candidates stand out because they have been shown to play a major role in melanisation patterns in insects including Lepidoptera. Melanisation in *Papilio glaucus* is caused by an unidentified sex-linked gene, and coincides with *Ddc* expression patterns but can also be down-regulated by *ebony* [15], [18]. *Ddc* and *PO* have been identified as key enzymes for cuticular melanisation in *Manduca sexta* [31], and in *Papilio xuthus*, *Ddc, punch* and *TH* are up-regulated in melanising patches of the larval cuticle [12], [16]. In *Drosophila melanogaster*, *yellow* has been implicated in melanin pattern formation on larval mouth parts and adult wings, mediated either through insertion of a transposable element closely downstream of *yellow* [32] or by a wing-specific *cis*-regulatory element [10]. The difference between the morphs in *B. betularia* could also be considered as variation in pattern rather than melanisation *per se* because the typical form has black patches, which define an underlying pattern, whereas *carbonaria* is completely black, except for two small white spots where the forewing attaches to the thorax. Thus the potential of *yellow* to regulate the distribution of melanin across wings and cuticle makes it a very relevant candidate gene. *Tan* is another promising melanisation candidate because it can counteract the reduction of Dopamine, a melanin substrate, by *ebony* [19].

To determine whether a candidate gene is likely to be directly involved in *B. betularia* melanisation, we tested whether polymorphisms in the candidate genes co-segregate with the melanic phenotype in a *carbonaria*-typical backcross. Polymorphisms within a candidate gene must be in full linkage disequilibrium within this cross if the gene is directly responsible, that is, the marker allele distribution should be identical to the phenotype distribution in the offspring. If a *cis*-regulatory element of a melanisation pathway gene causes the phenotypic switch, we would expect the allele distribution of the candidate gene to be closely similar to the phenotype distribution.

## Results and Discussion

Sixteen melanisation candidate genes, as indicated in Figure 1, were isolated either by targeted PCR (11 genes) or by a transcriptome approach (5 genes). They cover all of the core melanisation pathway genes with the possible exception of some members of the *yellow* family, and additionally include some of the immune response genes. All genes were identified based on a blastx search [33] against the GenBank database (www.ncbi.nlm.nih.gov). The best hits in GenBank are presented in Table 1. The *B. betularia* sequences are available in GenBank under accession numbers GU980199-GU980212, GU953216-GU953231, and GS923573. In principle, the ‘best hit’ was considered the ortholog but, additionally, the difference between the best hit and the second-best hit within a species and the proportion of positive amino acid (aa) matches were taken into account to confirm sequence identity. The existence of *B. betularia* specific paralogs of the melanisation genes cannot be entirely excluded, but is highly unlikely because recently derived paralogs are usually revealed through sequence polymorphisms that do not follow Mendelian segregation, i.e. variation that does not represent allelic variation within a gene. We did not encounter such patterns in any of the genes presented here, though it is the case that *Pap3* and the *yellows* belong to families known to have high duplication rates.

**Table 1:** NCBI blastx ‘best hits’.

Gene	*B. betularia* Accession #	Blast hit accession number	Blast hit species	Blast hit gene name	e-value	positives; gaps
*aaNAT*	GU953216	NP_001073122.1	*Bombyx mori*	arylalkylamine N-acetyltransferase	7e-112	236/261 (90%); 0
*Ddc*	GU953217	BAB68545.1	*Mamestra brassicae*	dopa decarboxylase	0.0	440/457 (96%); 0
*Dhpr*	GU953218	XP_001652256.1	*Aedes aegypti*	dihydropteridine reductase	4e-46	121/146 (82%); 0
*ebony*	GU953219	BAE43845.2	*Papilio xuthus*	ebony	1e-80	343/394 (87%); 1
*ferritin LC*	GU953220	AAF44717.1	*Manduca sexta*	ferritin	3e-41	168/182 (92%); 0
*ferritin HC*	GU953221	AAG41120.1	*Galleria mellonella*	26kDa ferritin subunit	4e-09	65/67 (97%); 0
*punch*	GU953222	BAH11149.1	*Bombyx mori*	GTP cyclohydrolase I isoform A	6e-95	184/194 (94%); 2
*henna*	GU953223	BAE66652.1	*Papilio xuthus*	phenylalanine hydroxylase	8e-92	167/172 (97%); 0
*Pap1*	GU953224	AAX18636.1	*Manduca sexta*	prophenoloxidase-activating proteinase-1	2e-151	305/379 (80%); 1
*Pap3*	GU953225	AAX18637.1	*Manduca sexta*	prophenoloxidase-activating proteinase-3	8e-32	85/115 (73%); 2
*Ppo1*	GU953226	NP_001037335.1	*Bombyx mori*	phenoloxidase subunit 1 precursor	0.0	424/469 (90%); 0
*Ppo2*	GU953227	ABM65701.1	*Heliothis virescens*	prophenoloxidase-2	0.0	617/690 (89%); 1
*tan*	GU953228	XP_001599569.1	*Nasonia vitripennis*	N/A (conserved hypothetical protein)	7e-79	233/361 (64%); 11
*TH*	GU953229	BAE43824.1	*Papilio xuthus*	tyrosine hydroxylase	0.0	538/561 (95%); 1
*yellow*	GU953231	NP_001037434.1	*Bombyx mori*	yellow-y	0.0	442/532 (83%); 26
*yellow2*	GU953230	NP_001037424.1	*Bombyx mori*	yellow2/yellow-fa	1e-145	289/349 (83%); 5

Blastx results for the 16 candidate genes in the GenBank database. The hits with the highest e-values are listed. The positives and gaps are listed to provide a context for the e-values because a low e-value does not necessarily reflect low similarity. The ‘Low complexity regions filter’ was disabled for the *henna* search. The *tan* ortholog of *Nasonia vitripennis* is not named, instead the *Apis mellifera* hit with slightly lower score confirms the identity of this gene (acc. # XP_623115.1; *Apis mellifera* tan; Expect  = 2e-74, Positives  = 235/353 (66%), Gaps  = 11/353 (3%)). *Yellow2* blastx reveals two identical *B. mori* sequences that have been named differently (NP_001037424.1  =  *yellow2*, ABC96695.1  =  *yellow-fa*).

The proportion of positive matches for *tan* relative to the ‘best hit’ were lower than for the rest of the genes (64%), but it is evident that the *B. betularia* sequence is the proper ortholog because *tan* has no paralogs in any of the examined insect genomes (species specific blast: *Bombyx mori, Tribolium castaneum, Apis melifera, D. melanogaster, Anopheles gambiae, Nasonia vitripennis*). The relatively low proportion of positive aa matches in *tan* is due to the moderately conserved nature of this gene in insects and not a result of mis-identification. The aa sequence of *tan* in different insect species is aligned in fasta-alignment Text S1. When these blast searches were performed the contiguous sequence of *tan* in *B. mori* was not deposited in any of the sequence databases and therefore had to be assembled from different sequences (fasta-alignment Text S2). More recently, however, the full sequence has become pubclicly available on GenBank.

The *Pap3* search revealed an unambiguous best hit in *Manduca sexta*, with *Pap2* and a number of hemolymph proteinases as alternatives within the same species, but with considerably less similarity. As *Pap3* belongs to the extensive serine proteinase family there is greater potential of hitting paralogs if the true ortholog is not in the database. This seems unlikely however, as the serine proteinases of *M. sexta* have been thoroughly surveyed [23], [34], [35], [36], [37], suggesting that they should be well covered in GenBank. The *yellow* genes also belong to a gene family, but this family is smaller and better characterized (at least in *B. mori*) than the serine proteinases [38]. The ortholog status of the two *B. betularia* genes based on aa sequence is unambiguous, but members of this gene family have not always been named consistently. The *B. betularia yellow* gene is known as *yellow* and *yellow-y* in *B. mori*; the *B. betularia yellow2* gene is named both *yellow2* and *yellow-fa* in *B. mori*.

*Ppo1* and *Ppo2* are much alike and have reciprocal saturated e-values (0.0), but the proportion of positive matches and presence of aa regions that are diagnostic allow them to be identified without question. The relatively low e-value for *ferritinHC* reflects the short length of the available *B. betularia* sequence (97 aa), but the similarity within this short stretch is very high and unambiguous. The remaining genes did not raise any issues because they have high e-values, a high percentage of positive matches and no obvious paralogs. This is also the case for *Pap1* because, unlike *Pap3*, it is a very distinct gene [22].

Polymorphisms were found for all the 16 genes in the father of the *carbonaria*/typical backcross. Most of these polymorphisms were inside introns, some in coding regions and one in a closely linked BAC-end sequence (Table S1). The genes *tan*, *TH* and *henna* displayed a hemizygous genotype distribution, with heterozygotes restricted to the males and one of either alleles in the individual females. This is consistent with the WZ/ZZ sex determination system that is generally found in Lepidoptera and it reveals that these three genes are on the Z-chromosome in *B. betularia*, which corresponds with their location in *B. mori*.

The segregation patterns of the polymorphisms in all of the 16 candidate genes were significantly different from the *carbonaria/*typical phenotype distribution, within a progeny sample of 38 *carbonaria* and 35 typicals (Table 2). This demonstrates, somewhat surprisingly, that none of the genes examined, or linked regulatory units are primarily responsible for the melanisation switch in *B. betularia*. As our study includes essentially all the structural gene candidates (with the possible exception of undetected members of the *yellow* family), the result suggests that the genetic switch controlling the *carbonaria* polymorphism is more likely to be a higher level *trans*-regulatory gene [39]. Pigmentation studies in drosophilids are revealing a diversity of mechanisms regulating variation in epidermal melanin deposition, both within and between species. Thus, variation in a transcription factor binding site in the *cis*-regulatory element of *yellow*, together with downregulation of *ebony*, mediate species-specific wing pattern melanisation [10]; and sequence variation either within or closely linked to *tan* and *ebony* underlie intraspecific polymorphism in body colour within *Drosophila americana* [40]. In other cases, pigment patterning appears to be effected through the altered expression of regulatory genes themselves, possibly through variation in enhancer sequences [39].

**Table 2:** Test of association between genetic variation in 16 melanisation gene candidates and offspring phenotype.

locus	allele A		allele B		*X* ^2^ value
	carb	typ	carb	typ	
*aaNAT*	15	20	23	15	0.516545
*Ddc*	14	21	21	17	0.533008
*Dhpr*	16	15	22	20	0.999927
*ebony*	21	19	17	16	0.999844
*ferritinHC*	18	17	20	18	0.999712
*ferritinLC*	18	17	20	18	0.999712
*henna*	16	20	22	15	0.648496
*punch*	16	14	22	21	0.998396
*Pap1*	20	15	18	20	0.873806
*Pap3*	18	17	13	25	0.182833
*Ppo1*	27	20	11	15	0.673701
*Ppo2*	28	19	10	16	0.393203
*tan*	20	20	18	15	0.98527
*TH*	20	20	18	15	0.98527
*yellow*	21	14	17	21	0.636792
*yellow2*	22	12	16	23	0.252829

Distribution of 38 *carbonaria* and 35 typical offspring among the paternal alleles (A and B) at each candidate locus.

In contrast to the success of the candidate gene approach applied to melanism in vertebrates, particularly through *Mc1r* [4], [5] but also tyrosinase-related protein 1 [41], *Agouti* [42], and *K* locus [43], the same strategy has been far less useful as a means to identifying polymorphisms controlling melanism in insects [44], [45]. A rare exception is for abdominal and thoracic trident pigmentation in *Drosophila melanogaster* which co-segregates with nucleotide variation in and around *ebony* [46], [47]. This is in spite of the fact that melanic patterns are frequently associated with differential expression of one or more genes from the same set of candidates: *ebony*, *tan*, *TH*, *Ddc* or *yellow* [10], [12], [15], [40], [47], [48]. The current impression that vertebrate melanism is frequently the result of variation within structural, rather than regulatory, genes, goes some way to explaining this apparent difference, as structural genes forming part of a biosynthetic pathway are easier to target. Why the regulatory machinery of pigment melanin production should be more complex or less conserved in insects is not obvious from a comparison of vertebrate and invertebrate melanogenesis [9], and it may be that a restricted set of conserved regulatory melanisation or patterning genes for insects will eventually emerge.

Other than ruling out specific candidate genes, the present study does not bring us any closer to finding the ‘*carbonaria* gene’. Having effectively exhausted the *a priori* list of promising melanisation candidates, we are currently in the process of constructing a linkage map of *B. betularia* to identify the region that controls this famous polymorphism. The *B. betularia*-specific coding sequences that were isolated for this candidate gene analysis will be useful in future gene expression studies, and the expanded set of degenerate primers (Table S2) serves as a resource for applying similar approaches to other insects.

## Materials and Methods

### Experimental design

The offspring of a cross between a homozygous typical female (daughter of a wild pairing collected in Morkery Wood, Linconshire, UK) and a heterozygous *carbonaria*/typical male (wild caught in Greasby, Cheshire, UK) were used to examine whether candidate genes coincide with the *carbonaria* phenotype. Caterpillars were reared on oak leaves and adult phenotypes were scored after eclosion of the pupae. All procedures were performed following our institutional animal husbandry guidelines. DNA of half a thorax was extracted with a standard phenol-chloroform procedure [49]. Sixteen genes were screened for co-segregation of polymorphisms with the *carbonaria* phenotype. Eleven of these were isolated using degenerate primer PCR with cDNA template, and sequences of the remaining five were generated as part of a *B. betularia* transcriptome sequencing project. Polymorphisms were usually obtained from introns since these are more likely to include genetic variation than exons. However, in the case of *henna*, for which no internal polymorphism could be found, the end-sequence of a BAC clone containing this gene was used as a closely linked marker for genotyping.

### Primer design

A number of the degenerate primers used were designed by Mitchell *et al.* [50] and some by Hartzer *et al.* [51], sometimes slightly modified; the remainder were newly designed (Table S2). Nucleotide sequences of orthologous candidate genes in Hexapoda were obtained from NCBI (www.ncbi.nlm.nih.gov), ButterflyBase (http://butterflybase.ice.mpg.de) and SilkDB (http://silkworm.genomics.org.cn) and aligned based on their aa sequences to identify conserved regions. Blast searches [33] were performed to identify paralogs. If present, highly similar domains of paralogs were aligned to the target gene alignments to make sure that only gene-specific, rather than gene-family specific, consensus regions were used for primer design. The primers were designed to cover synonyms of codon triplets over the full length of the recognition site, except for the *ferritinHC* primers which have a long 5′ region based on the *Bombyx mori* sequence and only a relatively short degenerate 3′ match within a wide range of insect species. All primers based on *B. betularia* specific sequence were designed with Oligo 6 [52]. Their names start with ‘Bb’ in Table S2. Introns were identified either by a trial and error approach with randomly placed primer pairs or with primers that were positioned based on the assumption of conserved intron position between *B. betularia* and *B. mori*. The intron positions in *B. mori* were identified by comparing coding sequence and genomic sequence from the SilkDB database.

### RNA extraction and cDNA synthesis for gene-targeting

Total RNA was extracted from half a thorax of an adult in 1 ml of Trizol (Invitrogen, Carlsbad, CA, USA) following the manufacturers protocol. The cDNA was synthesized from 1 µg total RNA with 200 units Superscript III reverse transcriptase (Invitrogen), 40 units RNAseOUT (Invitrogen), 1X first-Strand Buffer, 5 µM DTT and 50 pmol oligoT_18_, or 50 pmol M13-polyTv, or 2 pmol gene-specific primer (GSP) in a 20 µl reaction. Primer sequences are available in Table S2. The oligoT_18_-primed cDNA was generated to serve as template to obtain internal coding regions of target genes, the M13-polyTv-primed cDNA was used for 3′ RACE and the cDNA strands generated with GSPs were used for 5′ RACE. First strand transcription involved one hour at 50°C for oligoT_18_-primed and M13-polyTv-primed cDNA and one hour at 55°C for cDNA generated with GSPs. The reverse transcriptase was subsequently heat inactivated at 70°C for 15 min.

### Gene-specific sequence targeting

Internal regions were amplified with degenerate consensus primers based on gene regions conserved in a wide range of insects (cds 1^st^ fragment primers in Table S2). The initial internal sequence was sometimes extended with an additional degenerate primer outside the initial sequence and a *B. betularia*-specific primer from within (cds extension primers in Table S2), or alternatively, two additional degenerate primers targeting a non-overlapping fragment were used to cover more internal sequence (cds additional primers in Table S2). The gap between two non-overlapping fragments was bridged by PCR (cds bridging primers in Table S2). The sequences were extended by means of rapid amplification of cDNA ends (RACE). Some genes were completed in both directions using 5′ RACE and 3′ RACE, and others only in the 3′ direction with 3′ RACE. *FerritinHC* contained an informative polymorphism within the initial part of the sequence and was not extended any further in either direction. The initial, extended and additional internal regions were amplified in 15 µl reaction volumes containing 0.6 units AmpliTaq Gold (Applied Biosystems), 1X AmpliTaq buffer I, 0.35 µM of each primer, 0.2 mM of each dNTP, 0.5 µl oligoT_18_-primed cDNA. Cycling regime was 9 min 94°C, 11 cycles of 30s 94°C, 30s T_a_td, 50s 72°C, 30 cycles of 30s 94°C, 30s T_a_, 50s 72°C, with T_a_td either [55°C →50°C (−0.5°C per cycle)], [52°C →47°C (−0.5°C per cycle)] or [50°C →45°C (−0.5°C per cycle)] and T_a_  = 50°C, 47°C, 45°C respectively. PCR cycle details are provided per primer combination in Table S2.

PCRs were inspected on 2% agarose gels and if needed, the product of interest (based on expected size) was isolated with the Qiagen gel extraction kit (Qiagen GmbH, Hilden, Germany). The original concentration of the gel-extracted amplicons were restored by 10 PCR cycles with the same T_a_ and reaction mix as used for the previous PCR, except for the template, which was 2 µl gel-extract in this case. Gaps between segments of coding sequence were filled in with 15 µl PCRs using primers on either side of the gaps with 0.5 µl oligoT_18_-primed cDNA as template, 0.6 units AmpliTaq Gold, 1X AmpliTaq buffer I, 0.35 µM of each primer, 0.2 mM of each dNTP and the 35X57HS (hot-start) cycling conditions described in Table 3. The partial *B. betularia Ddc* sequence deposited in GenBank (accession number EU032788) was not available at the time these experiments were performed.

**Table 3:** PCR conditions for different experiments.

PCR experiment	polymerase	Initial denaturation	cycle step 1 denaturation	cycle step 2 annealing	cycle step 3 extension	number of cycles
35X57	Amplitaq	3 min 94°C	30 s 94°C	30 s 57°C	50 s 72°C	35
35X57HS	Amplitaq Gold	9 min 94°C	30 s 94°C	30 s 57°C	50 s 72°C	35
38X60HS	Amplitaq Gold	9 min 94°C	30 s 94°C	30 s 60°C	60 s 72°C	38
45X60HS	Amplitaq Gold	9 min 94°C	30 s 94°C	30 s 60°C	60 s 72°C	45
35X68LR	Advantage 2	1 min 95°C	15 s 95°C		6 min 68°C	35
21X68LR	Advantage 2	1 min 95°C	15 s 95°C		6 min 68°C	21

### 3′ RACE

The template used for 3′ RACE was M13-polyTv-primed cDNA, which has a synthetic extension of the poly-A tail that acts as a target for the M13 primer. The PCR primer combination consists of a forward GSP positioned in the internal sequence and the M13 primer. PCRs were performed in 15 µl reaction volumes containing 0.6 units AmpliTaq Gold, 1X Amplitaq buffer I, 0.35 µM of each primer, 0.2 mM of each dNTP, 0.5 µl M13-polyTv-primed cDNA and 38X60HS cycling profile (Table 3). A nested primer was used to sequence the PCR products and primer walking (PW) was used to extend the sequence towards the 3′ end for PCR products that were larger than a single reliable sequence read. The three consecutive GSPs used for 3′ RACE are specified as 3R_PCR, 3R_SEQ, and 3R_PW in Table S2 (although 3R_PW was only needed for a few genes).

### 5′ RACE

5′ RACE used cDNA that was primed with an antisense GSP. The gene-specific cDNA was cleaned with QIAquick PCR purification spin columns (Qiagen) to remove all dNTPs used in first-strand synthesis (to allow the construction of an uninterrupted G-tail subsequently). Then 5 µl of cleaned gene-specific cDNA was combined with 6.5 µl H_2_O, heated at 65°C for 5 min and chilled on ice immediately after. The 3′end of the cDNA (corresponding with the 5′ end of mRNA) was extended with a poly-G stretch by including the chilled cDNA in a 20 µl reaction mix containing 15 units Terminal deoxynucleotidyl transferase (TdT) (Invitrogen), 1X TdT buffer and 1 mM dGTP. The tailing reaction mix was incubated at 37°C for 30 min and heat inactivated for 3 min at 80°C. The G-tailed cDNA was PCR amplified with a mix of three primers. The first primer is M13_polyC, which has a 3′ poly-C region to match the synthesised poly-G tail and a 5′ M13 region to act as primer recognition site. The second primer is an M13 primer that acts as a forward primer once the M13_polyC is incorporated during the initial PCR cycles. The third primer is a gene-specific antisense primer that is nested relative to the first-strand synthesis primer. These nested GSPs are named 5R_PCR primers in Table S2. PCRs were performed in 15 µl containing 0.6 units AmpliTaq Gold (Applied Biosystems), 1X AmpliTaq buffer I, 0.15 µM M13_polyC primer, 0.35 µM of M13 primer, 0.35 µM of 5R_PCR primer, 0.2 mM of each dNTP and 2 µl G-tailed gene specific cDNA and 45X60HS cycling profile (Table 3). The PCR products were sequenced with nested sequencing primers, which are named 5R_SEQ primers in Table S2. *Pap1, Ppo2* and *yellow* required primer walking to obtain the full sequence, the primers used are named 5R_PW in Table S2.

### Transcriptome sequence

The transcriptome was constructed from a 5^th^ instar larva using the Clontech SMART™ PCR cDNA Synthesis Kit (Mountain View, CA, USA), following the ‘first-strand cDNA synthesis’ and ‘cDNA Amplification by LD PCR’ instructions. Total RNA was extracted in Trizol (as described above). The cDNA synthesis was performed for 1 hour at 42°C in 10 µl with 1X First-Strand Buffer, 200 units Superscript II, 1.2 µM 3′ SMART CDS Primer II A, 1.2 µM SMART II A Oligonucleotide, 2 mM DTT, 1 mM of each dNTP, and 0.5 µg total RNA. Amplification took place in 200 µl containing 1X Advantage 2 PCR Buffer, 0.2 mM of each dNTP, 0.24 µM 5′ PCR Primer II A and 1X Advantage 2 Polymerase Mix. PCR was performed with the 21X68LR program (Table 3). The transcriptome was sequenced on a 454 FLX Titanium (Roche, Branford, CT, USA) and assembled with Newbler software (Roche). A blastx search [33] against the *B. mori* annotated genes in SilkDB was used to identify the melanisation candidates.

### Localization of introns

Primers were positioned in presumed neighboring exons (based on the assumption that intron positions are conserved between *B. betularia* and *B. mori*) to amplify the intron between them. The intron in *punch* was obtained with a forward *B. betularia*-specific primer within partial cds from the transcriptome and a degenerate reverse primer within the next exon. *FerritinHC* required a trial-and-error primer-pair approach because the intron position in *B. betularia* differed from that in *B. mori*. With the exception of the *Pap1* intron amplification, PCRs were performed in 15 µl containing 0.6 units AmpliTaq (Applied Biosystems), 1X AmpliTaq buffer I, 0.35 µM of each primer, 0.2 mM of each dNTP and 25 ng of gDNA as template with 35X57 cycle conditions (Table 3). *PAP1* required a long-range PCR polymerase due to the large size of the target intron. The reaction was performed in 30 µl containing 1X Advantage 2 PCR Buffer, 0.2 mM of each dNTP, 0.21 µM of each primer, 1X Advantage 2 Polymerase Mix and 50 ng gDNA template with the 35X68LR two-step PCR conditions described in Table 3. *Dhpr* does not contain introns in *B. mori* and two proximal primers were included in this experiment to test whether introns are also absent in *B. betularia*.

### BAC identification

A 5X coverage BAC library, supplied with superpools and matrixpools for PCR-based BAC identification, was constructed by Amplicon Express (Pullman, WA, USA). DNA from the clone that was positive for *henna* was isolated with the BACMAX™ DNA Purification Kit (Epicentre Biotechnologies, Madison, WI, USA). End sequences were obtained by Sanger sequencing with T7 Promoter Primer and BAC-R primer (primer sequences in Table S2).

### Genotyping

The parental sequences were screened for single nucleotide polymorphisms (snps) and for insertion-deletions (indels) that were heterozygous in the *carbonaria* father of the cross and homozygous in the typical mother. These polymorphisms give male-informative (MI) backcross segregation in the offspring. PCR-RFLP was used to assay snps within restriction endonuclease recognition sites for *ferritinHC* (*Taq*I), *TH* (*Mse*I), *ebony* (*HpyCH4*IV) and *PpoI* (*Hae*III), whereas *ferritinLC* contains an indel that is large enough to distinguish the two alleles unambiguously on a 2% agarose gel. Offspring genotypes at the remaining polymorphic loci were established by Sanger sequencing.

### Genotype-phenotype co-segregation

Co-segregation between the *carbonaria* phenotype and each of the candidate gene′s genotypes was assessed by simple inspection and more formally using a chi-square test. To account for stochastic deviations from an exact 1∶1 ratio of paternal alleles in the offspring, for each candidate gene locus, the expected values for *carbonaria* versus typical offspring were calculated with respect to the actual frequencies of each paternal allele (A or B) in the offspring sample. This test is conservative because the confidence value refers to whether the association between genotype and phenotype is larger than would be expected by chance. In reality, given the single locus dominant nature of the *carbonaria* polymorphism, we expect a 100% match between offspring phenotype and genotype for a marker locus tightly linked to the functional polymorphism, and a marginally weaker association if linkage is weaker (e.g. *cis*-regulatory element).

## Supporting Information

Text S1 Insect amino acid alignment of tan.
(0.00 MB TXT)

Text S2 Assembly of tan coding sequence in *Bombyx mori* based on different resources. The tan sequence for *Bombyx mori* is wrongly predicted in SilkDB and Kaikobase and the sequences in ButterflyBase and SilkBase are incomplete. This alignment assembles tan for *B. mori* using the combined information in these databases and a 3'RACE sequence produced especially for this assembly.
(0.01 MB TXT)

Table S1 Table of polymorphisms used to genotype the melanisation candidates.
(0.05 MB DOC)

Table S2 Table of primers used to isolate and genotype the candidate genes.
(0.21 MB DOC)
