# Supplementary material for: Industrial Melanism in the Peppered Moth Is Not Associated with Genetic Variation in Canonical Melanisation Gene Candidates
Source: PLoS One. 2010 May 28;5(5):e10889. doi: 10.1371/journal.pone.0010889 (PMC2878321; doi:10.1371/journal.pone.0010889)
Supplement: Table S1 — Table of polymorphisms used to genotype the melanisation candidates. (0.05 MB DOC) [file pone.0010889.s003.doc]

| locus | Genbank accession number | Position from start | polymorphism | Location of  Polymorphism | Comments |
| --- | --- | --- | --- | --- | --- |
| *aaNAT* | GU980199 | 162 bp | C/T snp | exon |  |
| *Ddc* | GU980200 | 623 bp | A/G snp | intron |  |
| *Dhpr* | GU953218 | 218 bp | A/G snp | exon | Dhpr has no introns |
| *ebony* | GU980201 | 214 bp | A/G snp | intron |  |
| *ferritinLC* | GU980202 | 408 bp | indel | intron |  |
| *ferritinHC* | GU980203 | 328 bp | A/G snp | intron |  |
| *punch* | GU980204 | 130 bp | A/T snp | intron |  |
| *henna*/41K14 | GS923573 | 219 bp | A/T snp | non-coding | Closely linked BAC-end |
| *Pap1* | GU980205 | 243 bp | A/C snp | intron |  |
| *Pap3* | GU980206 | 74 bp | A/G snp | intron |  |
| *Ppo1* | GU980207 | 374 bp | C/G snp | intron |  |
| *Ppo2* | GU980208 | 302 bp | G/T snp | intron |  |
| *tan* | GU980209 | 642 bp | A/G snp | intron |  |
| *TH* | GU980210 | 144 bp | G/T snp | intron |  |
| *yellow* | GU980212 | 232 bp | G/T snp | intron | Intron in 5’UTR |
| *yellow2* | GU980211 | 93 bp | C/T snp | exon |  |
