# Supplementary material for: Industrial Melanism in the Peppered Moth Is Not Associated with Genetic Variation in Canonical Melanisation Gene Candidates
Source: PLoS One. 2010 May 28;5(5):e10889. doi: 10.1371/journal.pone.0010889 (PMC2878321; doi:10.1371/journal.pone.0010889)
Supplement: Table S2 — Table of primers used to isolate and genotype the candidate genes. (0.21 MB DOC) [file pone.0010889.s004.doc]

| Target region | Primer name | Primer sequence | Primer origin | PCR conditions |
| --- | --- | --- | --- | --- |
| **arylalkylamine N-acetyltransferase (*aaNAT*)** | | | | |
| cds 1^st^ fragment | aaNAT_238dgF1 | TTYTTYCGNGAYGARCC | 3 | 55🡺50  touchdown |
|  | aaNAT_589dgR1 | ADICCYCTNCCYCTCCA | 3 |  |
| 3’RACE | Bb_aaNAT_3R_PCR | GTTGCAGCGGTGGACGAGAATG | 3 | 38X60HS |
|  | Bb_aaNAT_3R_SEQ | AAGTCCGAAGAGTGCCCGAACC | 3 | NA |
| 5’RACE | Bb_aaNAT_5R_FS | TCCTGACTTCCAGCACGCTGTG | 3 | NA |
|  | Bb_aaNAT_5R_PCR | CTCCGGAAGTTTCTCGAAGATT | 3 | 45X60HS |
|  | Bb_aaNAT_5R_SEQ | GATGACAATTCCGACATAGTCT | 3 | NA |
| genotyping | Bb_aaNAT_293U | TGCCGAATCACACCAGACGA | 3 | 35X57 |
|  | aaNAT_82L (S) | GATGACAATTCCGACATAGTCT | 3 |  |
| **Dihydropteridine reductase (*Dhpr*)** | | | | |
| genotyping | Bb_Dhpr_12U | GACCTCAGTAAGCAAGCCGACC | 3 | 35X57 |
|  | Bb_Dhpr_433L (S) | CTTGGCAAGTTACTCGACAATCA | 3 |  |
| **Dopa decarboxylase (*Ddc*)** | | | | |
| cds 1^st^ fragment | Ddc19sF | ATGHTNGAYTGGYTVGGYCARATG | 1 | 50🡺45  touchdown |
|  | Ddc4sRC_bist | GGMATCTGCCAGTGGCGGTARTC | 1 mod |  |
| cds extension | DdcUpstrDG | GCGAARGCNATGACYGAYTAYAT | 3 | 55🡺50  touchdown |
|  | Bb_Ddc379L | GATGTAGTGCCCAACGTAGC | 3 |  |
| 3’RACE | Bb_Ddc_3R_PCR | CGCCTTGGACGAGATTGCAG | 3 | 38X60HS |
|  | Bb_Ddc_3R_SEQ | CCGCGATGTGGCTCAAGGAG | 3 | NA |
| genotyping | Bb_Ddc_127U | AAATGTAACTCGCCGCAGGT | 3 | 35X57 |
|  | Bb_Ddc_152L (S) | CACGCCGGACATGACGACTC | 3 |  |
| **NBAD synthase (*ebony/BAS*)** | | | | |
| cds 1^st^ fragment | ebonyPx_814U | CGGTTGGTATTGGTCCCGAC | 3 | 50🡺45  touchdown |
|  | ebonyPx_1710L | TCCTACCGTCTCGAAGAGGACC | 3 |  |
| 3’RACE | Bb_ebony_3R_PCR | CCGCGGCAGTCCAGAAATCC | 3 | 38X60HS |
|  | Bb_ebony_3R_SEQ | AACACATCGAGGGCAGCTTG | 3 | NA |
|  | Bb_ebony_3R_PW | CGCCCTATCAGTCAGAGCAG | 3 | NA |
| 5’RACE | Bb_ebony_5R_FS | CCTTCTGCAGCGTGCCGTAA | 3 | NA |
|  | Bb_ebony_5R_PCR | CCATCACTTCCGTGCTGCCG | 3 | 45X60HS |
|  | Bb_ebony_5R_SEQ | TGTGACCGCTCGCGCTGAAG | 3 | NA |
| genotyping | Bb_ebony_350U | GCCTCTAGCTTCTGCGATAA | 3 | 35X57 |
|  | Bb_ebony_598L | CTGGCTGCAGCGTCACGAAC | 3 |  |
| ***ferritin heavy chain*** | | | | |
| cds 1^st^ fragment | ferritin_F | CGCGAGCACGCGACCAAGC  TCATTGAMTAYCTKCTNATG | 3 | 55🡺50  touchdown |
|  | ferritin_R2 | CCGAGGAGTTTCTTGTCGAA  GATRAAYTCNCC | 3 |  |
| genotyping | Bb_ferritinHC_144U | GGCCTGCGAAAGCGATCCAG | 3 | 35X57 |
|  | Bb_ferritinHC_216L | CGCGCTGTCCCTTGTATTGC | 3 |  |
| ***ferritin light chain*** | | | | |
| genotyping | Bb_Frtn-LC_334U | GAAGGAGTTGGCCGAACGTGCTTT | 3 | 35X57 |
|  | Bb_Frtn-LC_445L | ATTTCGCGAATGGTCTTAGCTTGG | 3 |  |

| Target region | Primer name | Primer sequence | Primer origin | PCR conditions |
| --- | --- | --- | --- | --- |
| **GTP cyclohydrolase I (*GTPCHI/punch*)** | | | | |
| intron | Bb_GTPCHI_423U | GATCCCACCAGAGATGGTCT | 3 | 55🡺50  touchdown |
|  | Bb_GTPCHI_dgR | CGCACATTGAAAACATCTCG  ATRTCCTTAACNACCACCATYTCRTC | 3 |  |
| genotyping | Bb_GTPCHI_483U | TTCACAAAGGGATACGACCAAAG | 3 | 35X57 |
|  | Bb_GTPCHI_750L | CGAAAAGTTTTCTCAGTGGTTGCCATACTTAGT | 3 |  |
| **Phenylalanine hydroxylase (*PAH/henna*)** | | | | |
| BAC  screening | Bb_Henna 33U | CTGCTGTCGTCGCGTGACTT | 3 | 35X57 |
|  | Bb_Henna 339L | TACTGCAGCTCGCCGAAAGA | 3 |  |
| genotyping | 41K14ups_121U (S) | CACTGAGTGCCACAAGCTACATT | 3 | 35X57 |
|  | 41K14ups_503L | GGTCAAATATTTACAATTGGCCTACCAT | 3 |  |
| **Prophenoloxidase activating proteinase 1 (*Pap1*)** | | | | |
| cds 1^st^ fragment | PAP1_592F | GAYGARTTYCCNTGGATG | 3 | 55🡺50  touchdown |
|  | PAP1_1255_LEP_R | TAWACKCCTGGCCAKCCRTC | 3 |  |
| 3’RACE | Bb_PAP1_3R_PCR | CGTGGAAGTTGGCAGCGATGTTT | 3 | 38X60HS  NA |
|  | Bb_PAP1_3R_SEQ | GTCAACAAATACCAGACGCTGAG | 3 |  |
| 5’RACE | Bb_PAP1_5R_FS | ATGGGCATGCCGAGCTTTAGTTT | 3 | NA |
|  | Bb_PAP1_5R_PCR | TGGGTGAGGGACGGCTACTT | 3 | 45X60HS |
|  | Bb_PAP1_5R_SEQ | TGAGGGACGGCTACTTGTACTGG | 3 | NA |
|  | Bb_PAP1_5R_PW | CGCGGGTCTGGAGTCATCATCGTG | 3 | NA |
| Intron | Bb_PAP1_2U | TATCAATGCGGTGGCGTGTT | 3 | 35X68LR |
|  | Bb_PAP1_225L | TGAGGGACGGCTACTTGTACTGG | 3 |  |
| genotyping | Bb_PAP1_176U | TGGAAACTCGGCAGCCTGTTAAT | 3 | 35X57 |
|  | Bb_PAP1_681L | GCGGAGGGTCAGCACAGTTATT | 3 |  |
| **Prophenoloxidase activating proteinase 3 (*Pap3*)** | | | | |
| Intron | Bb_PAP3_48U | CGATCTGCTTGCCAACGATAGA | 3 | 35X57 |
|  | Bb_PAP3_356L | GTCTCCTTTGCAGGAATCTTTGCC | 3 |  |
| genotyping | Bb_PAP3_250U | TGATCTCTCATGCGTTGCTCTAAT | 3 | 35X57 |
|  | Bb_PAP3_356L | as above | 3 |  |
| **Pro-phenoloxidase 1 (*Ppo1*)** | | | | |
| cds 1^st^ fragment | PpoI_F | CAYCAYTGGCAYTGGCAYTTGG | 2 mod | 50🡺45  touchdown |
|  | PpoI_R | GCCAGCGGTAGAACACSGGGT | 3 |  |
| cds  additional | Pi_PPO1_f4 | ACCCSGTGTTCTACCGCTGGC | 2 | 52🡺47  touchdown |
|  | Pi_PPO1_r2 | CTCNGGGTACTTGCGGTC | 2 |  |
| cds  bridging | Bb_PpoI_gapF | TGGAGACTTGGCGCGACCGT | 3 | 35X57 |
|  | Bb_PpoI_gapR | GCGACGCACCGTGTTGTTACCA | 3 |  |
| 3’RACE | Bb_PpoI_3R_PCR | ACCTTCCGCGACCAATCAGC | 3 | 38X60HS |
|  | Bb_PpoI_3R_SEQ | CGGATGCGGCTGGCCTCACC | 3 | NA |
| Intron | Bb_PpoI_115U | TACAACGTAGAGCGCATGTG | 3 | 35X57 |
|  | Bb_PpoI_303L | TCTATAGCGGCCATGAAACGGTC | 3 |  |
| genotyping | Bb_PpoI_704U | GCCACCCAGGTCTGTGCCTAATG | 3 | 35X57 |
|  | Bb_PpoI_303L | TCTATAGCGGCCATGAAACGGTC | 3 |  |

| Target region | Primer name | Primer sequence | Primer origin | PCR conditions |
| --- | --- | --- | --- | --- |
| **Pro-phenoloxidase 2 (*Ppo2*)** | | | | |
| cds 1^st^ fragment | PpoIIdeg_659 | ATCATYCCAAGRGAYTAYAC | 3 | 52🡺47  touchdown |
|  | PpoIIdeg_1217_21L | GAARCTGTGVCCGTTGTTRTG | 3 |  |
| cds  additional | PpoIIdeg_1217_21U | CAYAACAACGGBCACAGYTTC | 3 | 55🡺50  touchdown |
|  | PpoIIdeg_1865_23L | GGCCABCCGCADCCGCAGWARTT | 3 |  |
| cds  bridging | Bb_PpoII_454U | CGCAGCAACTAACCATCGAC | 3 | 35X57 |
|  | Bb_PpoII_647L | TCCATGCGTGCCAGCGGTAG | 3 |  |
| 3’RACE | Bb_PpoII_3R_PCR | CCCGGACAAGCGAGCGATGG | 3 | 38X60HS |
|  | Bb_PpoII_3R_SEQ | CAACCGAGGCCAATCCAAGG | 3 | NA |
| 5’RACE | Bb_PpoII_5R_FS | CCATGCGTGCCAGCGGTAGA | 3 | NA |
|  | PpoII_5R_PCR | AGCGAACCATGCCAGTAGAG | 3 | 45X60HS |
|  | PpoII_5R_SEQ | GTGCGTTGTTCAGGCGTTCA | 3 | NA |
|  | PpoII_5R_PW | GAGATGCCAATGCCAGTTGTG | 3 | NA |
| genotyping | Bb_PpoII_30U | GAGCACCGACTGGCCTACTG | 3 | 35X57 |
|  | Bb_PpoII_197L (S) | GTGCGTTGTTCAGGCGTTCA | 3 |  |
| **NBAD hydrolase (*tan*)** | | | | |
| cds 1^st^ fragment | TANdg85b | CAYTAYGARGTNGGATTCGA | 3 | 50🡺45  touchdown |
|  | TAN_dg_454a | GACATHATRTARAARTGATT | 3 |  |
| 3’RACE | Bb_TAN_3R_PCR | GCCAACATGAAAAAGAAGTTCCC | 3 | 38X60HS |
|  | Bb_TAN_3R_SEQ | ATGACCTCATCGGCAACGTAAAC | 3 | NA |
|  | Bb_TAN_3R_PW | TTGTAATTTGTACCAACGCATAA | 3 | NA |
| intron | Bb_TAN_617U | AGAGAATTCTCAGTGACGACGGT | 3 | 35X57 |
|  | Bb_TAN_962L | AATAGTATTGATAACGGCGTCCT | 3 |  |
| genotyping | Bb_TAN_281U (S) | ATTCGGTGGCCAACGCAGTT | 3 | 35X57 |
|  | Bb_TAN_742L | ATGCAGACGCGGGCTACGAT | 3 |  |
| **Tyrosine hydroxylase (*TH/pale*)** | | | | |
| cds 1^st^ fragment | ThLep1compF | CTACAGCATYGAGAACGGYTA | 3 | 55🡺50  touchdown |
|  | ThLep_748L | TTGTCCSCGAAACCGGGATG | 3 |  |
| cds  additional | ThLep_1120U | CGTCACGCCAACTCGCCYTTC | 3 | 55🡺50  touchdown |
|  | ThLep_1632L | ACGGCRTTGGTYAGGTGCAGCATC | 3 |  |
| cds  bridging | THgap_F | AACTACAATGGTTGCGTTGAGC | 3 | 35X57 |
|  | THgap_R | GAATGCAGTCAGGTTCGGGTGT | 3 |  |
| 3’RACE | Bb_TH_3R_PCR | CCTCTGATCCGGAAATCGAA | 3 | 38X60HS |
|  | Bb_TH_3R_SEQ | AGCCCGCAAGCACTTCCGTA | 3 | NA |
| 5’RACE | Bb_TH_5R_FS | TTCGGTCAGGGTATAATCGT | 3 | NA |
|  | Bb_TH_5R_PCR | CCAACTTTGCAGCGTCGTCC | 3 | 45X60HS |
|  | Bb_TH_5R_SEQ | GGGAGGTGTCGCTAGTGCCA | 3 | NA |
| genotyping | Bb_TH_1096U | CCTCTGATCCGGAAATCGAA | 3 | 35X57 |
|  | Bb_TH_1272L | TGGTAAGGCTGTACGGAAGT | 3 |  |

| Target region | Primer name | Primer sequence | Primer origin | PCR conditions |
| --- | --- | --- | --- | --- |
| ***yellow*** | | | | |
| cds 1^st^ fragment | yellow_637dgF3 | GAYGARYTGGGMTAYGG | 3 | 50🡺45  touchdown |
|  | yellow_1009dgR2 | CCGACIGCRTTYTGRTC | 3 |  |
| 3’RACE | Bb_yellow_3R_PCR | CAAGTCCTGGAGATTCAGTC | 3 | 38X60HS |
|  | Bb_yellow_3R_SEQ | TGCGGCACTTCCTATGAAACCAC | 3 | NA |
|  | Bb_yellow_3R_PW | CGCGGCTAGTATAATCTCATACA | 3 | NA |
| 5’RACE | Bb_yellow_5R_FS | TCTGTATCCATCAGTGCCAATA | 3 | NA |
|  | Bb_yellow_5R_PCR | CTCCCCATTGGAAGTTGAGA | 3 | 45X60HS |
|  | Bb_yellow_5R_SEQ | GGACGGGGAAGAAATAGCTG | 3 | NA |
|  | Bb_yellow_5R_PW | TGAGGGAATATGGGCACACGTT | 3 | NA |
| genotyping | Bb_yellow_1U | AGTCTAATTCGAGCTCTCGC | 3 | 35X57 |
|  | Bb_yellow_164L (S) | GATGGCCTGCTGCTTCGAGT | 3 |  |
| ***yellow2*** | | | | |
| cds 1^st^ fragment | Bm_yellow-fa/fb_355dgF1 | CARTAYAAYAAYGTGCC | 3 | 52🡺47  touchdown |
|  | Bm_yellow-fa_949dgR2b | AADATICCRTCNCTCCA | 3 |  |
| 3’RACE | Bb_yellow2_3R_PCR | CATGGAGAGGACGCCGAATC | 3 | 38X60HS |
|  | Bb_yellow2_3R_SEQ | GATGGTGGACACCGGCCTAC | 3 | NA |
| Intron | Bb_yellow2_394U | TCCAACGCCTTCGCCTACATACCA | 3 | 35X57 |
|  | Bb_yellow2_551L | GGGCTATACTGAAAATTCCGTCGC | 3 |  |
| genotyping | Yellow-2_genot-F | AGCTGTCAGTCAAGTCCGGTCA | 3 | 35X57 |
|  | Bb_yellow2_551L (S) | as above | 3 |  |
| **Miscellaneous primers** | | | | |
|  | oligoT_18_ | TTTTTTTTTTTTTTTTTT | universal |  |
|  | M13-polyTv | CAGGAAACAGCTATGACCT(17)V | universal |  |
|  | M13-polyC | CAGGAAACAGCTATGAC(19) | universal |  |
|  | M13 | CAGGAAACAGCTATGACC | universal |  |
|  | SMART II™ A Oligonucleotide | AAGCAGTGGTATCAACGCAGAGTACGCGGG | clontech |  |
|  | 3' SMART™ CDS Primer II A | AAGCAGTGGTATCAACGCAGAGTACT(20)VN | clontech |  |
|  | 5' PCR Primer II A | AAGCAGTGGTATCAACGCAGAGT | clontech |  |
|  | T7 Promoter Primer | TAATACGACTCACTATAGGG | universal |  |
|  | BAC-R | CTCGTATGTTGTGTGGAATTGTGAGC | universal |  |

Primer pairs are categorized as follows: ‘cds 1st fragment’ primers were used to generate the initial coding sequence, ‘cds extension’ primers produced a partially overlapping sequence relative to the initial sequence, ‘cds additional’ primers amplified part of the coding sequence that did not overlap with the initial sequence, ‘cds bridging’ primers filled the gap between initial and additional sequence. Primer origin column: 1 = designed by [1], 2 = designed by [2], 3 = primers designed specifically for this study. The primers that were used as sequencing primers to genotype the family are indicated with (S).

1. Mitchell A, Mitter C, Regier JC (2000) More Taxa or More Characters Revisited: Combining Data from Nuclear Protein-Encoding Genes for Phylogenetic Analyses of Noctuoidea (Insecta: Lepidoptera). Syst Biol 49: 202-224.

2. Hartzer KL, Zhu KY, Baker JE (2005) Phenoloxidase in larvae of *Plodia interpunctella* (Lepidoptera: Pyralidae): molecular cloning of the proenzyme cDNA and enzyme activity in larvae paralyzed and parasitized by *Habrobracon hebetor* (Hymenoptera: Braconidae). Arch Insect Biochem Physiol 59: 67-79.
